# Work-related sexual and gender harassment: conceptual challenges and the need for evidence-based prevention

**DOI:** 10.5271/sjweh.4121

**Published:** 2023-10-01

**Authors:** Ida EH Madsen, Britt D Nielsen

**Affiliations:** 1 National Research Centre for the Working Environment, Copenhagen, Denmark; 2 National Institute of Public Health, Copenhagen, Denmark

The topic of work-related sexual harassment has gained renewed attention in recent years following the public accounts of sexually harassing experiences – many of which were work-related – that emerged during the #MeToo movement beginning in October 2017 (1). The many testimonials from the movement illustrated the magnitude of the problem and put faces to the numbers of statistics on the topic. However, work-related sexual harassment is not a new phenomenon. In fact there are accounts of experiences that could be classified as sexual harassment in legal procedures dating back to at least the early 1900s (2). In labelling these experiences, feminist legal scholars MacKinnon (3) and Farley (4) introduced the term 'sexual harassment' in the late 1970s in the United States. The behaviors were considered unlawful when occurring in a work-setting and conducted or condoned by the employer. Since they negatively affected employment conditions of women, they were considered a violation of laws regarding equal rights between women and men (5). From the outset, sexual harassment was considered a legal rather than a working-conditions problem. Over time, it has become acknowledged also in the literature on working conditions, and recent studies have illustrated the salience of this exposure, showing increased risk of severe outcomes such as long-term sickness absence (6), depression (7), psychotropic treatment (8), alcohol-related morbidity and mortality (9), suicide and suicide attempt (10).

## Definitions and concepts

But how should we define this exposure, which appears to be associated with such severe negative consequences? This question has been a topic of long-standing academic debate (11, 12). Legally, sexual harassment definitions differ between countries. In the European Union legal framework, sexual harassment is when “*Any form of unwanted verbal, non-verbal or physical conduct of a sexual nature occurs, with the purpose or effect of violating the dignity of a person, in particular when creating an intimidating, hostile, degrading, humiliating or offensive environment*.” (13). From an academic perspective, leading scholars have argued that research should not be confined to those behaviors that are illegal but should have a broader focus and definition of the concept (14). An early definition from pioneering researcher Fitzgerald and colleagues (15) was that sexual harassment is “*Unwanted sex-related behavior at work that is appraised by the recipient as offensive, exceeding her resources, or threatening her wellbeing”*. Another more recent perspective focuses on sex-based rather than sexual harassment, suggesting that “*sexual harassment should be viewed as harassment that is based on sex – as behavior that derogates, demeans, or humiliates an individual based on that individual’s sex*” (16, p644). A key notion to this research is that sexual harassment is rooted in power and gender dynamics rather than sexual attraction.

A leading conceptual framework has been the tripartite model, introduced in the mid 1990s (17), stating that sexual harassment consists of three interrelated but distinct phenomena: gender harassment, unwanted sexual attention, and sexual coercion (5, 15). Gender harassment denotes behaviors that convey insulting, hostile or degrading attitudes about another person’s gender (often, but not always, towards women). The behaviors do not necessarily involve any explicit sexual content, and they are not aimed at sexual encounters. Unwanted sexual attention comprises sexual behaviors and advances – verbal or nonverbal – that are unwelcome, offensive, and unreciprocated. According to the tripartite model, this category also encompasses sexual assault, such as rape. Sexual coercion relates to quid pro quo scenarios, such as being promised a reward in exchange for sex or threatened with sanctions if one does not comply. The tripartite model has been foundational to much research in the area, and proponents of the model argue that gender harassment is the most common but often overlooked form of sexual harassment (5). As an illustration, an iceberg model has been developed, proposing that the behaviors most commonly associated with sexual harassment – unwanted sexual advances – are merely the visible tip of a much larger structure, with the hidden base of the iceberg consisting of a wide array of derogatory treatment of women (5). Most of the empirical work supporting the model, however, has been conducted in the US, and often analyzes data collected a couple of decades ago. Consequently, it is unclear how accurately the model describes the phenomenon of sexual harassment in a contemporary context outside of the US. More recently, a number of studies concerning the potential serious consequences of sexual harassment have emerged, mainly from a Scandinavian context (6–10). Notably, most of these studies are based on questionnaires measuring sexual harassment using a single item – a method referred to as the self-labelling approach (18). This measurement identifies only those cases of sexual harassment that are defined by the respondent as such. Research suggests that the estimates for prevalence are much higher when measuring situations that could be classified as sexual harassment – but may not be labelled as such by the respondent – using specific questions concerning experiences related to unwanted sexual attention, gender harassment, or sexual coercion (19).

## Theoretical perspectives on the causes of sexual harassment

Similar to debates concerning the definition and measurement of sexual harassment, there is no single unifying theory to explain why sexual harassment occurs. According to Pina et al (11), the most widely recognized theories include: (i) sociocultural theories; (ii) organizational theories; and (iii) natural-biological theories. In brief, the sociocultural theories, largely feminist [eg (20),], view gender inequality and sexism as the root of sexual harassment. Similarly, organizational theories [eg (21),] acknowledge that power differentials play a central role in sexual harassment. However, organizational theories do not focus on gender-specific differentials, but on status inequalities within the organization. These theories also consider other organizational factors, such as tolerance of sexual harassment, gender norms, and organizational policies. These theories are often supported by empirical studies showing that especially women, young individuals, and individuals in precarious work arrangement are more often exposed to sexual harassment (22). In contrast, the natural-biological theories [eg (23),] view sexual harassment as an expression of a natural sexual attraction between people. According to this understanding, the reason that men are more inclined to sexually harass than women is because they have a higher sex drive than women. Considering that each of these perspectives may be too simplistic to understand the phenomenon of sexual harassment, the four-factor model (24) has been developed. This model can be characterized as multifactorial, seeking to incorporate many of the elements from the preceding theories. According to this model, sexual harassment depends on (i) a motivation to harass (eg, any combination of power, control, or sexual attraction), (ii) overcoming internal inhibitions (eg, moral restraints), (iii) overcoming external inhibitions (eg, specific organizational workplace barriers), and (iv) target resistance (eg, assertiveness or the targets’ relative status within the workplace).

## Workplace prevention

The prevention of work-related sexual harassment is a topic of high importance and – perhaps unsurprisingly – high complexity. To our knowledge, no interventions exist that have strongly documented effects on reducing the risk of sexually harassing behaviors at work. Researchers often highlight sexual harassment policies and training as essential prevention efforts, but the evidence of their effect remains limited (25–28). The aim of preventive workplace efforts is typically to change employee’s knowledge, skills, and attitudes, for instance to increase self-protection skills, alter gender beliefs and reduce tolerance of sexual harassment in order to change the organizational culture. However, despite more than a decade of research, rigorous evaluations remain scarce and most existing studies are from the US, possibly with limited generalizability (22, 25, 27, 28). Training interventions are diverse, ranging from brief online programs to intensive face-to-face programs that include group discussions and roleplay (27, 28). Training may include all employees or target specific groups with a special responsibility to manage sexual harassment, eg, managers and human resource personnel. Given the diversity of the interventions, it is difficult to assess how the duration, content, and mode of delivery affects the intervention's effectiveness. Similarly, considerable controversy exists regarding zero-tolerance policies – a widely applied instrument (25, 29). Proponents argue that zero-tolerance policies send a strong signal that reduces employee’s proclivity to sexually harass others and encourages targets and witnesses to come forward. However, critics argue that strict zero-tolerance policies that are followed by severe punishment may make targets and witnesses more hesitant to come forward and result in pushback from employees if sanctions and procedures are deemed unfair (29).

## Future directions in research and practice

To advance our state of knowledge in this complex area of sexual harassment, we suggest two important tasks. First, there is a need for conceptual clarity. Given that even the definition of the concept of sexual harassment has been a topic of debate for decades (11, 12), this is no small task. Nonetheless, it is pivotal to advancing the state of knowledge that we strive towards conceptual clarity so that it is clear what phenomenon we are actually researching or trying to prevent. We argue that it may be helpful to use specific terminology whenever possible, and, to this end, the terminology of the tripartite model, distinguishing between gender harassment, unwanted sexual attention and coercion may be useful. The usefulness of insisting on using the umbrella term 'sexual harassment' to encompass all of these very different experiences may be more questionable. Furthermore, whether the tripartite model provides an accurate understanding of sexual harassment in countries outside the US, and in a contemporary context, is an empirical question that remains unanswered. Second, the lack of solid evidence-based methods to prevent work-related sexual harassment is striking. In our experience, the recent attention on sexual harassment in public debate has spawned an extensive demand from workplaces asking what to do in terms of prevention. Currently, there is little to offer regarding evidence-based preventive interventions. Thus, an important task for future research is to develop and test interventions so we may provide answers to inform prevention. Such development and testing may also be guided by appropriate use of specific terminology so that it is clear what behaviors the interventions are designed to prevent. Given that sexual harassment is a substantial work environment problem associated with severe health consequences, its prevention constitutes an important societal task.
